# Concurrent Guillain–Barre/acute transverse myelitis overlap syndrome after COVID-19 infection in a patient with ITP: A case report

**DOI:** 10.1097/MD.0000000000040346

**Published:** 2024-11-08

**Authors:** Ruomeng Chen, Liang Wang, Binbin Wang, Xiujuan Song, Xiaoyun Liu

**Affiliations:** a Department of Neurology, The First Hospital of Hebei Medical University, Shijiazhuang, Hebei Province, China; b Department of Neurology, The Second Hospital of Hebei Medical university, Shijiazhuang, Hebei Province, China.

**Keywords:** acute transverse myelitis, COVID-19, GBS/ATM overlap syndrome, Guillain–Barre syndrome, ITP

## Abstract

**Rationale:**

Patients with chronic immune diseases, such as idiopathic thrombocytopenic purpura (ITP), should be alert for Guillain–Barre/acute transverse myelitis (GBS/ATM) overlap syndrome after infection with coronavirus disease 2019 (COVID-19).

**Patient concerns:**

A 65-year-old male with an ITP history, who presented with limb numbness and weakness, urinary retention, right peripheral facial paralysis, and diplopia 2 weeks after being diagnosed with COVID-19.

**Diagnosis:**

GBS/ATM overlap syndrome secondary to COVID-19.

**Interventions:**

Five days intravenous immune globulin, methylprednisolone (500 mg) was added for treatment. He was discharged with medicine and continued to take Methylprednisolone tablets (60 mg/d), Eltrombopag olamine (25 mg 1/d), Mecobalamine tablets, vitamin B1, and rehabilitation treatment outside the hospital.

**Outcomes:**

The patient significantly improved after initial treatment, he returned to normal life after 8 weeks. Five months later, he was infected with COVID-19 for the second time, exhibiting only symptoms of upper respiratory tract infection and no other discomfort.

**Lessons:**

COVID-19 infection can lead to secondary myelitis and GBS, and GBS/ATM overlap syndrome is rare, but patients are significantly better after immunization and hormone therapy.

## 1. Introduction

The coronavirus disease 2019 (COVID-19) pandemic bagan in late 2019, which is caused by severe acute respiratory syndrome coronavirus 2 (SARS-CoV-2). SARS-CoV-2 is an enveloped, single-stranded RNA β-coronavirus. Spikes on the virus surface formed by S protein enter target cells by binding to human Angiotensin-Converting Enzyme 2 (ACE2) receptors. In addition to its presence in cardiovascular, kidney, and testis, ACE2 expression has also been detected in neurons and neurogliocyte.^[1]^ Therefore, COVID-19 may lead to neurological complications, and some cases of Guillain–Barre syndrome (GBS) or acute transverse myelitis (ATM) associated with COVID-19 infection have been reported. During the COVID-19 epidemic, the morbidity of GBS increased about 5.41 times,^[2]^ mostly acute inflammatory demyelinating polyneuropathy (AIDP) type,^[3]^ and ATM accounted for 1.2% of COVID-19-related neurological complications.^[4]^ However, GBS/ATM overlap syndrome associated with COVID-19 is rare. We present a case of Guillain–Barre/acute transverse myelitis (GBS/ATM) overlap syndrome after COVID-19 infection in an idiopathic thrombocytopenic purpura (ITP) patient.

## 2. Case report

On January 8, 2023, a 65-year-old male patient with a history of ITP was admitted to the neurological department due to “progressive limb numbness and weakness for 7 days.” Fourteen days before the onset of symptoms, he was infected with COVID-19 according to the results of rapid antigen test based on colloidal gold immunochromatographic assay for the detection of COVID-19 infection. At first, the symptoms were fatigue and numbness in both hands and feet. Over the next 48 hours, it gradually aggravated, the symptoms developed from the distal end of the limb to the proximal end and caused difficulty in walking, holding heavy objects, and using chopsticks. One day later, he had difficulty urinating and defecating, so an indwelling catheter was placed. Two days later, he developed right facial paralysis and diplopia. Magnetic resonance imaging (MRI) brain revealed brain atrophy and white matter lesions (Figure S1, Supplemental Digital Content, http://links.lww.com/MD/N850), and treatment did not show improvement in the local hospital. For further diagnosis and treatment, he was admitted to our hospital. On the neurological examination, he had peripheral facial paralysis on the right side, bilateral upper and lower limbs can resist partial resistance along with no bowel sensation. His reflexes were biceps (++), triceps (+), redial (+), bilateral knee and ankle reflex (0), and without pathological extensor plantar responses bilaterally. Hypoalgesia and deep sensations loss in bilateral lower limbs below the knee, the coordination movement was not coordinated.

**Figure 1.**
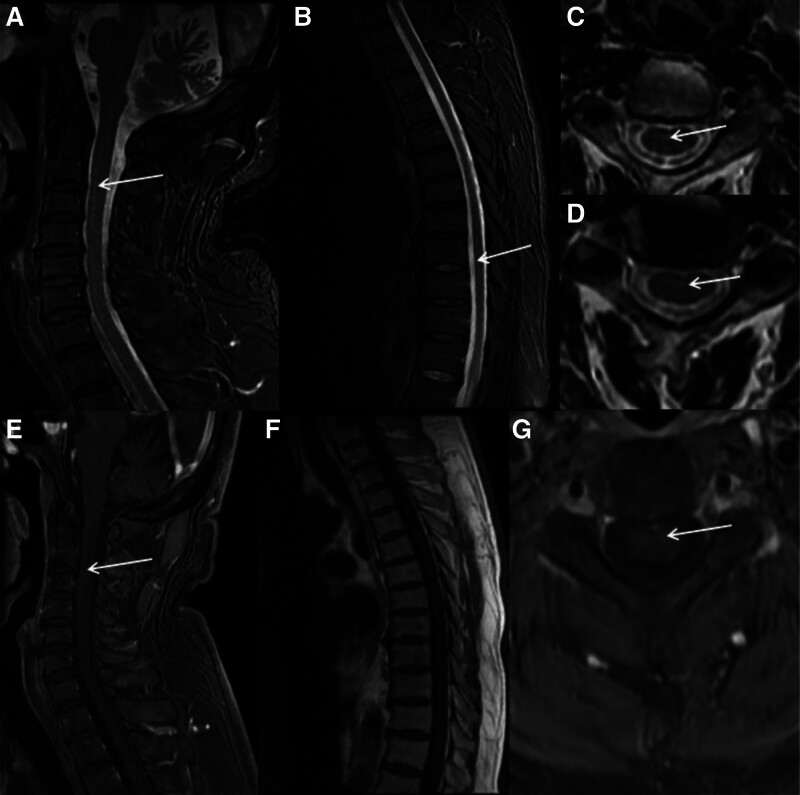
The sagittal T2 image (A and B) shows abnormal hyperintense signal from C2 to C3 and T11. The coronal T2 images (C) shows abnormal hyperintense signal at cervical cord. Slight enhancement signal in the enhanced sequence (E–G). No significant enhancement signal in the thoracic spinal cord enhanced sequence (F).

Considering the initial clinical diagnosis was Guillain–Barre syndrome, the patient was treated with intravenous immunoglobulin (IVIg) on the second day of admission, at the same time laboratory investigations (Table 1), MRI, and electromyography (EMG) were done. Spinal MRI showed T2WI shows abnormal hyperintense signal from C2 to C3 and T11. Slight enhancement signal was found in the enhanced sequence of cervical spinal cord (Fig. 1). EMG revealed reduced amplitude of multiple peripheral motor nerves and no abmormality of sensory nerves (Table 2). In consideration of his history, a chest computed tomography scan (CT scan) was performed and showed interstitial pneumonia with “ground glass” opacities (Figure S2, Supplemental Digital Content, http://links.lww.com/MD/N850), suggestive of coronavirus disease (COVID-19). Cerebrospinal fluid (CSF) showed an albuminocytologic dissociation, and detection of peripheral ganglioside antibodies by immunoblotting test presented anti-Sulfatide antibody IgG (+), anti-GM1 antibody IgG (+), anti-GM3 antibody IgG (+) (Table S1, Supplemental Digital Content, http://links.lww.com/MD/N849, Figure S3, Supplemental Digital Content, http://links.lww.com/MD/N850). According to the auxiliary examinations, the patient was considered for a diagnosis of Guillain–Barre syndrome (Acute Motor Axonal Neuropathy, AMAN)/acute myelitis overlap syndrome, so methylprednisolone (500 mg) was added for treatment. On the 10th day of admission, the numbness of the patient’s limbs improved and he regained bowel sensation and could eat by himself with chopsticks. On the 12th day of admission, he could urinate, defecate and stand but could not ambulate alone, so he was discharged with medicine and continued to take Methylprednisolone tablets (60 mg/d), Eltrombopag olamine (25 mg 1/d), Mecobalamine tablets, and vitamin B1 outside the hospital. He persisted in rehabilitation treatment and could walk about 50 meters independently after 4 weeks, and returned to normal life after 8 weeks. Three months after discharge, spinal MRI was reexamined, showing no abnormally high signal (Figure S4, Supplemental Digital Content, http://links.lww.com/MD/N850). Five months after discharge, the patient contracted COVID-19 for the second time, exhibiting only symptoms of upper respiratory tract infection and no other discomfort.

**Table 1:** Investigations.

Investigations		Results	Normal reference range
Blood laboratory findings	White blood cell count	10.18 × 10^9^/L	3.5–9.5 × 10^9^/L
	Neutrophil	7.14 × 10^9^/L	1.8–6.3 × 10^9^/L
	Platelet	257 × 10^9^/L	125–350 × 10^9^/L
	hs-CRP	9.3 mg/L	0–6 mg/L
	Sodium	135.7 mmol/L	137–157 mmol/L
	Folic acid	3.08 ng/mL	>3.38 ng/mL
	Vitamin B12	>2000 pg/mL	211–911
	Toxoplasma IgM	45.56 AU/mL	0–10 AU/mL
	Erythrocyte sedimentation rate	20 mm/h	<15 mm/h
	Immunoglobulin G	26.9 g/L	7.5–15 g/L
	Antinuclear antibody	1:320	<1:100
	Mitochondrial antibody typing assay	(+)	(−)
	AQP4	(−)	(−)
	MBP	(−)	(−)
	MOG	(−)	(−)
	GQ1b	(−)	(−)
	Ganglioside antibody profile	Anti-Sulfatide IgG (+) Anti-GM1 IgG (+) Anti-GM3 IgG (+)	(−)
Cerebrolspinal fluid	Cranial pressure	120 mmH_2_O	80–180 mmH_2_O
	CSF protein	1.05 g/L	0.15–0.45 g/L
	CSF glucose	6.11 mmol/L	2.5–4.5 mmol/L
	CSF cell count	2 × 10^6^/L	0-8 × 10^6^/L
	Cytology	Lymphocyte response is dominant	/
	Next-generation sequencing	Negative	

AQP4 = aquaporin-4 antibody, CSF = cerebrospinal fluid, GQ1b = Ganglioside Q1b antibody, hs-CRP = high-sensitive C reactive protein, MBP = myelin basic protein antibody, MOG = myelin oligodendrocyte glycoprotein antibody.

**Table 2:** Electromyogram results.

Motor nerve conductions		Latency (mS)	Amplitude (mV)	Conduction velocity (m/s)
Ulnar nerve (left)	Wrist-abductor digiti minimi	2.59	6.7	
	Below elbow-wrist	7.10	6.2	57.6
Ulnar nerve (right)	Wrist-abductor digiti minimi	2.28	8.0	
	Below elbow-wrist	7.51	7.0	53.5
Median nerve (left)	Wrist-abductor policis brevis	2.87	2.7↓	
	Below elbow-wrist	6.39	2.3↓	56.8
Median nerve (right)	Wrist-abductor	3.14	6.2	
	Below elbow-wrist	7.61	5.4	49.2
Tibial nerve (left)	Med.malleolus-abd. hallucis b	4.55	11.3	
	Popliteal fossa-med.malleolus	13.1	7.8	43.3
Tibial nerve (right)	Med.malleolus-abd. hallucis b	3.92	3.7↓	
	Popliteal fossa-med.malleolus	12.8	3.0↓	41.7
Peroneal nerve (left)	Ankle-extensor digit.Brevis	6.57↑	2.7↓	
	Caput fibulae-ankle	13.4	2.3↓	43.9
Peroneal nerve (right)	Ankle-extensor digit.Brevis	4.92	1.97↓	
	Caput fibulae-ankle	13.2	1.72↓	36.2↓
Facial nerve-buccal branches (left)	Mastoid-orbicularis oris muscle	2.35	1.45	
Facial nerve-buccal branches (right)	Mastoid-orbicularis oris muscle	3.02	1.76	
Facial nerve-zygomatic branches (left)	Ear-zy	2.98	1.33	
Facial nerve-zygomatic branches (right)	Ear-zy	3.06	1.55	
Sensory nerve conductions		Latency (mS)	Amplitude (mV)	Conduction velocity (m/s)
Ulnar nerve (left)	V finger-wrist	1.90	16.2	60.5
Ulnar nerve (right)	V finger-wrist	1.87	16.5	61.5
Median nerve (left)	II finger-wrist	2.27	18.2	59.5
Median nerve (right)	II finger-wrist	2.30	16.2	60.9
Sural nerve (left)	Calf-Lat.malleolus	2.21	10.6	54.3
Sural nerve (right)	Calf-Lat.malleolus	2.19	9.5	54.8
F-wave		F-lat (ms)	M-lat (ms)	F-wave occurrence rate
Ulnar nerve (left)	Wrist-ADM	25.2↑	2.5	100%
Ulnar nerve (right)	Wrist-ADM	19.0	2.4	100%
Median nerve (left)	Wrist-APB	29.2↑	3.0	70%
Median nerve (right)	Wrist-APBhallucis b	26.7	3.0	100%
Tibial nerve (left)	Med. malleolus-abd. hallucis b	47.9	4.8	100%
Tibial nerve (right)	Med. malleolus-abd. hallucis b	49.2	4.2	100%
Sural nerve (left)	Ankle-EDB	54.6	5.8	100%
Sural nerve (right)	Ankle-EDB	56.6↑	5.8	70%

“↓”represents a decrease in amptlitude or conduction velocity;“↑”represents an increase in latency.

## 3. Discussion

The patient in our case had been infected with COVID-19 14 days before the illness onset. In this case, the main clinical manifestations of the patient were: limb numbness and weakness, urinary retention, right peripheral facial paralysis, and diplopia; physical examination showed decreased muscle strength, peripheral facial paralysis, and ataxia; ancillary investigations indicated motor axonal injury, myelitis, CSF albuminocytologic dissociation, and positive ganglioside antibody. Therefore, the presence of both acute motor axonal neuropathy and acute myelitis was considered. After being treated with steroids and IVIg, the clinical symptoms significantly improved.

Positive ganglioside antibodies are rare in patients with GBS/ATM overlap syndrome, but the coexistence of 3 kinds of antibodies were observed in our patient. Ganglioside and sulfatide antibodies are closely associated with the development of autoimmune-mediated acute and chronic polyneuropathies. Serum anti antibody testing can help the diagnosis of GBS. Previous studies showed that anti-Sulfatide antibodies are associated with several subtypes of peripheral neuropathy, predominantly sensory or sensorimotor axonal neuropathies are most common,^[5]^ which may also exist in AMAN.^[6]^ Fan et al proposed that IgG anti-GM1 antibodies are associated with AMAN, and IgM antibodies of GM1, GM2, and GM3 are associated with facial nerve palsy in Chinese patients.^[7]^ Although GM1 antibodies are not specific for GBS, GM1 antibodies can be elevated in patients with AMAN, and anti-GM1 and GM3 antibodies may be associated with facial nerve palsy. Combined with the patient’s clinical manifestations, electromyographic findings, and cerebrospinal fluid findings, the patient can be diagnosed with GBS.

GBS/ATM overlap syndrome secondary to COVID-19 is rare, especially in an ITP patient. There were no cases of concurrent ITP with GBS/ATM overlap syndromes retrieved. The initial symptoms of GBS/ATM overlap syndrome are usually limb weakness, which gradually progresses to difficulty urinating or defecating, and respiratory failure in about 43.5%. The imaging findings showed high spinal cord T2WI signal, physical examination showed pathological signs (+) (29.4%), most of the cerebrospinal fluid albumin cytological dissociation, positive ganglioside antibodies are rare in patients with GBS/ATM overlap syndrome, but the coexistence of 3 kinds of antibodies were observed in our patient. Acute axonal polyneuropathy accounted for more than half of GBS/ATM overlap syndrome, and most of them were AMSAN.^[8]^ However, GBS/ATM overlap syndrome associated with COVID-19 main were AMAN. Spinal cord lesions are essential for GBS/ATM overlap syndrome. Steroids alone are not a good choice for the treatment of patients with GBS/ATM overlap syndrome, and steroids combined with IVIg or plasma exchange may be a better option. Although GBS or ATM generally have a good prognosis, some patients have a poor prognosis when they exist together.^[9]^ Some patients regained independent walking within 6 weeks.^[10–13]^ Our patient presented sensory disturbance, and had difficulty in urinating and defecating, 3 antibodies coexist, returned to normal life after 8 weeks after treatment with IVIg and methylprednisolone.

## 4. Conclusion

We conclude that GBS and ATM may occur simultaneously but is very rare, which is difficult for doctors to diagnose. When GBS patients present with sphincter disorder, sensory plane, and pyramidal tract signs, they should be alert to combine with acute myelitis. EMG and MRI play a crucial role in diagnosis. Spinal MRI is the key to the identification of acute myelitis, and the combination of IVIg and steroids is the most common treatment. Our patient with GBS/ATM overlap syndrome occurred after COVID-19 infection and had a history of ITP, which is very rare situation and deserves clinical attention.

## Acknowledgments

I would like to thank all my team members who have helped me to develop the fundamental and essential academic competence.

## Author contributions

**Data curation:** Xiujuan Song.

**Supervision:** Xiaoyun Liu.

**Writing – original draft:** Ruomeng Chen.

**Writing – review & editing:** Liang Wang, Binbin Wang.

## Supplementary Material


