# Supplementary material for: Concurrent Guillain–Barre/acute transverse myelitis overlap syndrome after COVID-19 infection in a patient with ITP: A case report
Source: Medicine (Baltimore). 2024 Nov 8;103(45):e40346. doi: 10.1097/MD.0000000000040346 (PMC11556959; doi:10.1097/MD.0000000000040346)
Supplement: Supplementary file 1 [file medi-103-e40346-s001.doc]

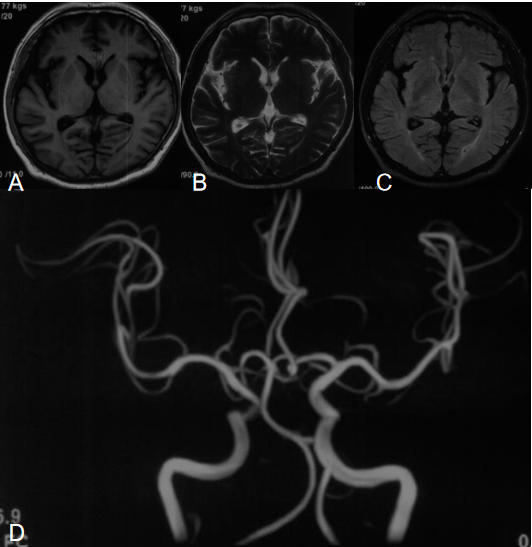


**Fig.S1**MRI of brain shows brain atrophy and FLAIR sequences shows white matter lesions(C);MRA suggests no intracranial vascular stenosis(D).


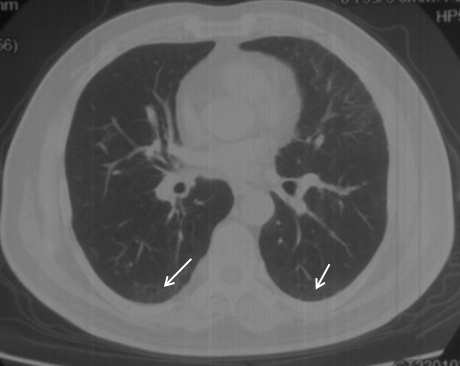


**Fig.S2**CT chest scans showed interstitial pneumonia with multiple "ground-glass" opacities in the right(A).


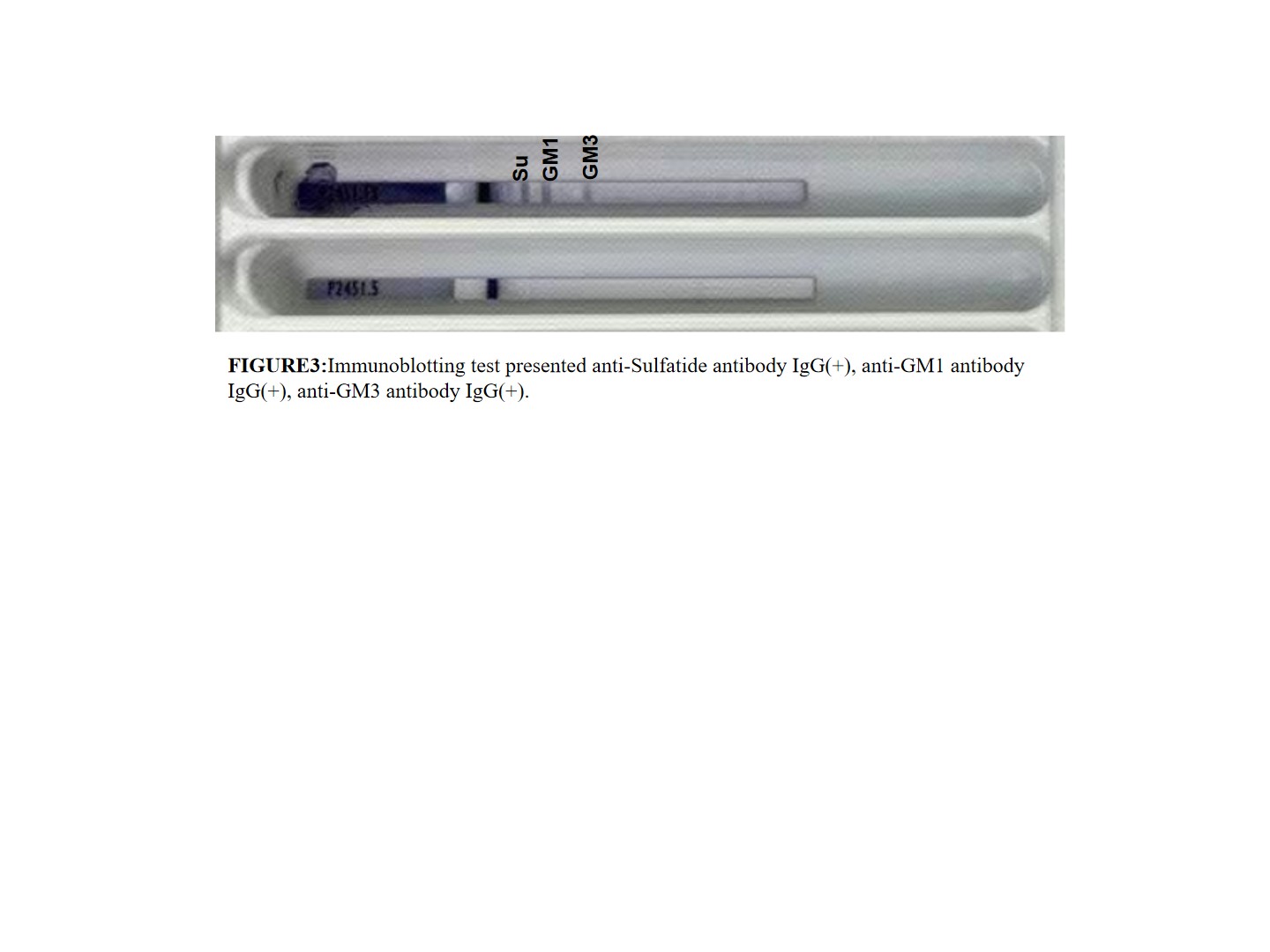


**Fig.S3**Immunoblotting test presented anti-Sulfatide antibody IgG(+), anti-GM1 antibody IgG(+), anti-GM3 antibody IgG(+).


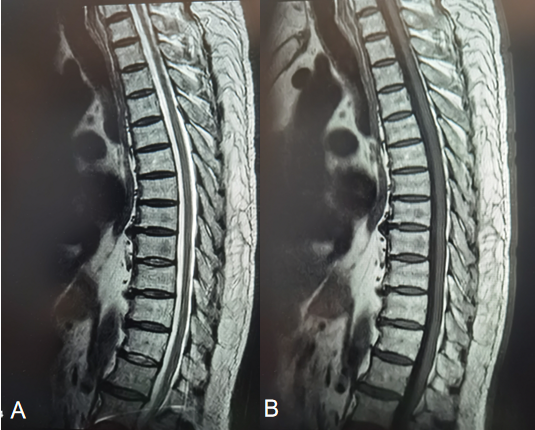


**Fig.S4**After 3 months, spinal MRI was reexamined, T11 abnormally high signal was missing.
