# Supplementary material for: Concurrent Guillain–Barre/acute transverse myelitis overlap syndrome after COVID-19 infection in a patient with ITP: A case report
Source: Medicine (Baltimore). 2024 Nov 8;103(45):e40346. doi: 10.1097/MD.0000000000040346 (PMC11556959; doi:10.1097/MD.0000000000040346)
Supplement: Supplementary file 2 [file medi-103-e40346-s002.docx]

| **TableS1** Peripheral neuropathy antibody, ganglioside antibody generalized spectrum test results | | | |
| --- | --- | --- | --- |
| **Investigations** | **Detection Methods** | **Results** | **normal reference range** |
| Anti-Sulfatide Antibody IgG | immunoblotting | (+) | （-） |
| Anti-GM1 Antibody IgG | immunoblotting | (+) | (-) |
| Anti-GM2 Antibody IgG | immunoblotting | (-) | (-) |
| Anti-GM3 Antibody IgG | immunoblotting | (+) | (-) |
| Anti-GM4 Antibody IgG | immunoblotting | (-) | (-) |
| Anti-GD1a Antibody IgG | immunoblotting | (-) | (-) |
| Anti-GD1b Antibody IgG | immunoblotting | (-) | (-) |
| Anti-GD2 Antibody IgG | immunoblotting | (-) | (-) |
| Anti-GD3 Antibody IgG | immunoblotting | (-) | (-) |
| Anti-GT1a Antibody IgG | immunoblotting | (-) | (-) |
| Anti-GT1b Antibody IgG | immunoblotting | (-) | (-) |
| Anti-GQ1b Antibody IgG | immunoblotting | (-) | (-) |
| Anti-Sulfatide Antibody IgM | immunoblotting | (-) | (-) |
| Anti-GM1 Antibody IgM | immunoblotting | (-) | (-) |
| Anti-GM2 Antibody IgM | immunoblotting | (-) | (-) |
| Anti-GM3 Antibody IgM | immunoblotting | (-) | (-) |
| Anti-GM4 Antibody IgM | immunoblotting | (-) | (-) |
| Anti-GD1a Antibody IgM | immunoblotting | (-) | (-) |
| Anti-GD1b Antibody IgM | immunoblotting | (-) | (-) |
| Anti-GD2 Antibody IgM | immunoblotting | (-) | (-) |
| Anti-GD3 Antibody IgM | immunoblotting | (-) | (-) |
| Anti-GT1a Antibody IgM | immunoblotting | (-) | (-) |
| Anti-GT1b Antibody IgM | immunoblotting | (-) | (-) |
| Anti-GQ1b Antibody IgM | immunoblotting | (-) | (-) |
| Peripheral blood was collected from the patients for ganglioside antibody profiles and the results are shown in the table above,(+) means the result is positive, (-) means the result is negative. | | | |
